# Prevalence of depression and associated symptoms among patients attending primary healthcare facilities: a cross-sectional study in Nepal

**DOI:** 10.1186/s12888-024-05794-0

**Published:** 2024-05-14

**Authors:** Nagendra P. Luitel, Bishnu Lamichhane, Pooja Pokhrel, Rudrayani Upadhyay, Tatiana Taylor Salisbury, Makhmud Akerke, Kamal Gautam, Mark J. D. Jordans, Graham Thornicroft, Brandon A. Kohrt

**Affiliations:** 1 Department of Global Public Health, Karolinska Institutet, Stockholm, Sweden; 2 Research Department, Transcultural Psychosocial Organization (TPO) Nepal, Baluwatar, Kathmandu Nepal; 3 Center for Global Mental Health Equity, Department of Psychiatry and Behavioural Health, George Washington University, Washington, D.C USA; 4 Centre for Global Mental Health, Health Service and Population Research Department, Institute of Psychology, Psychiatry & Neuroscience, King’s College London, London, UK; 5 Centre for Implementation Science, Health Service and Population Research Department, Institute of Psychology, Psychiatry & Neuroscience, King’s College London, London, UK

**Keywords:** Depression, Screening and detection, Treatment, Primary care, mhGAP, Nepal

## Abstract

**Background:**

Depression is a prevalent mental health condition worldwide but there is limited data on its presentation and associated symptoms in primary care settings in low- and middle-income countries like Nepal. This study aims to assess the prevalence of depression, its hallmark and other associated symptoms that meet the Diagnostic and Statistical Manual (DSM-5) criteria in primary healthcare facilities in Nepal. The collected information will be used to determine the content of a mobile app-based clinical guidelines for better detection and management of depression in primary care.

**Methods:**

A total of 1,897 adult patients aged 18–91 (63.1% women) attending ten primary healthcare facilities in Jhapa, a district in eastern Nepal, were recruited for the study between August 2, 2021, and March 25, 2022. Trained research assistants conducted face-to-face interviews in private spaces before the consultation with healthcare providers. Depression symptoms, including hallmark symptoms, was assessed using the validated Nepali version of the Patient Health Questionnaire (PHQ-9).

**Results:**

One in seven (14.5%) individuals attending primary health care facilities in Jhapa met the threshold for depression based on a validated cut-off score ( > = 10) on the PHQ-9. The most commonly reported depressive symptoms were loss of energy and sleep difficulties. Approximately 25.4% of women and 18.9% of men endorsed at least one of the two hallmark symptoms on the PHQ-9. Using a DSM-5 algorithm (at least one hallmark symptom and five or more total symptoms) to score the PHQ-9, 6.3% of women and 4.3% of men met the criteria for depression. The intra-class correlation coefficient for PHQ-9 total scores by health facility as the unit of clustering was 0.01 (95% confidence interval, 0.00-0.04).

**Conclusion:**

Depression symptoms are common among people attending primary healthcare facilities in Nepal. However, the most common symptoms are not the two hallmark criteria. Use of total scores on a screening tool such as the PHQ-9 risks overestimating the prevalence and generating false positive diagnoses. Compared to using cut off scores on screening tools, training health workers to first screen for hallmark criteria may increase the accuracy of identification and lead to better allocation of treatment resources.

## Introduction

Depression is a significant global health issue, particularly in low- and middle-income countries (LMICs) where the majority of people with depression live [1]. However, it often goes unnoticed in these countries. To address this, the task-sharing approach has been proposed [2], which involves training non-specialist healthcare providers to deliver mental health interventions in community settings. The World Health Organization (WHO) has developed the mental health gap action program (mhGAP) and implementation guide [3] to support this approach, which has been successfully implemented in over 90 countries [4]. Despite this, the detection rate of mental disorders by trained primary healthcare providers remains low, both in LMICs [5] and high-income countries [6].

In Nepal, only 24% of depression cases were detected by trained primary healthcare workers immediately after mhGAP-based training [7]. This raises concerns about the effectiveness of integrating mental health services into primary healthcare systems, especially considering that depression is a common condition in primary care [8]. To improve detection rates, routine screening for depression in primary care has been shown to be effective [9]. Various screening tools, such as the Patient Health Questionnaires (PHQ-9, PHQ-2) and the WHO Well-Being Index (WHO-5), have been recommended for use in primary care. However, their sensitivity in cross-cultural settings has not been widely evaluated [10]. Additionally, using PHQ-9 as a universal screener may not be feasible in LMICs due to limited resources. Using a mobile app-based clinical guide could be a potential strategy to enhance the detection of depression in primary care. Moreover, the severity of the individual item of PHQ-9 could help to determine the content of the mobile application because the DSM-PHQ algorithm closely aligns with the functionality of the app.

The purpose of this paper is to examine the prevalence of depression, its hallmark symptoms (depressed mood and anhedonia), and other related symptoms (e.g., fatigue, worthlessness, sleep disturbances) that meet Diagnostic and Statistical Manual (DSM-5) criteria in primary healthcare facilities in Nepal. The paper also seeks to identify factors associated with depression in order to estimate the target population in need of clinical services. Furthermore, the paper will investigate the most frequently reported symptoms of depression to inform the development of a mobile app-based clinical guideline for improved detection and management of depression in primary care.

## Methods

### Setting

This study was conducted as part of the Emilia (E-mhGAP Intervention guide in Low and middle-income countries: proof-of-concept for Impact and Acceptability) project, funded by UK Medical Research Council. The project aims to develop and test the feasibility and acceptability of a mobile-app-based clinical guide to improve the detection of depression in primary care [11]. The mobile app provides healthcare providers with the necessary information to assess, treat, and follow- up with individuals with depression. It follows the same protocol and decision trees as the paper version of the WHO mhGAP-IG V2 [12].

This study was a population-based cross-sectional health facility survey conducted prior to training primary health care workers in mobile app-based clinical guidelines. It was conducted between August 2, 2021 and March 25, 2022 in Jhapa, a district in eastern Nepal. The total population of Jhapa district is 998,054, with females accounting for more than half (52.1%) [13]. Nepal is one of the poorest countries in South-Asia, ranking 143rd out of 191 countries on the United Nations’ Human Development Index [14]. The country has a total population of approximately 29.1 million with 6, 666, 937 households.

In Nepal, Community Health Units (CHUs), Basic Health Service Centers (BHCs) in rural areas and Urban Health Centers (UHCs) in urban areas serve as the initial point of contact for basic health services. Health Posts (HPs) are the next level in the health care system. The third tier of health care consists of Primary Health Care Centers (PHCCs), which are higher- level facilities established in each electoral area as the first referral point. The municipal and district hospital are the highest-level healthcare institution within a district. The District Public Health Office (DPHO) or District Health Office (DHO) is responsible for coordinating health care activities in a specific district area [15]. There are 6 hospitals, 4 PHCCs, 42 HPs, 5 CHUs, 18 UHCs, 61 BHSCs in Jhapa district [16]. The study was conducted in two municipal hospitals, three PHCCs and five HPs. These health facilities offer primary healthcare services under the local government’s control. These health facilities were selected based on factors such as patient flow, accessibility, reasonable travel distance and availability of internet connectivity and electricity supply.

### Sample size and sampling

The study was conducted with randomly selected adults who attended primary health care facilities during the data collection period. The sample size was determined to allow the detection of change in diagnosis of depression in the primary health facilities between the baseline and subsequent follow-up studies. The sample size was determined based on previous data regarding primary care service utilization and depression screening rates [7]. We aim to screen approximately 50% of adult patients in primary care, with the potential to increase this percentage depending on patient flow. Our plan is to screen around 200 patients per arm per month, totaling 400 patients in the 1-month pre-training enrollment period and 1200 patients per country in the 3-month post-training enrollment period. This sample size will allow us to detect a 43% increase in the clinical case identification rate within each arm using the e-mhGAP-IG, with 90% power at a 5% significance level, assuming an intra-class correlation coefficient of 0.02 [11].

The inclusion criteria for participation in the study were: 18 years of age or above, fluent in Nepali language, time and availability to complete full survey which was administered orally by research assistants, and willingness to provide informed consent. Those who were incapable of providing informed consent because of an acute medical cause were excluded from the study.

We invited all eligible individuals at the health facility to participate in the study. The inclusion criteria for participation were being 18 years or older, fluent in Nepali, residents of selected municipalities/rural municipalities, and able to provide informed consent. We interviewed all eligible adults who entered into the health facilities and randomly selected one participant when multiple individuals were present simultaneously. Field research assistants created a list of eligible participants upon entering the clinic and then randomly selected a participant by drawing a name from the list using a piece of paper. Interviews were conducted with the selected participant before their consultation. Due to low client flow caused by COVID-19 restrictions, with only one participant visiting at a time, most participants were recruited individually without the need for randomization. Exclusion criteria included the inability to provide informed consent or currently experiencing an acute medical issue. Field research assistants conducted interviews with the consenting participants while they were waiting for health-care services.

### Instruments

The nine-item Patient Health Questionnaire (PHQ-9), a widely used tool for assessing depression, was used to assess depression [17]. Participants score nine common symptoms of depression based on their experience over the previous 2 weeks. It has a 4-point rating scale that ranges from 0 ‘not at all’ to 3 ‘always’. The first two items are the depression hallmark symptoms (depressed mood and anhedonia). At least one of these symptoms is required according to the DSM-5 to make a diagnosis of major depressive episode. The remaining seven items on the PHQ-9 are associated symptoms (e.g., fatigue, worthlessness, sleep disturbances). To meet DSM-5 criteria on the PHQ-9, at least one hallmark symptoms is required and 5 of the 9 total symptoms are required. The PHQ-9 has been culturally adapted, translated, and validated in Nepal [18]. The validation study determined that sum score cutoff of ≥ 10 had sensitivity = 0.94, specificity = 0.80, positive predictive value (PPV) = 0.42, negative predictive value (NPV) = 0.99, positive likelihood ratio = 4.62 and negative likelihood ratio = 0.07 when compared with a diagnosis of depression made using the Composite International Diagnostic Interview (CIDI) [18].

### Data collection

A two-and-a-half-week training was provided to nine field research assistants for data collection. The training focused on the basics of structured interviewing, study population, sample size and sampling procedure. The training also focused on instruments, scoring, referral system and inclusion/exclusion criteria. Various pre-tests and mock interviews were conducted during the training period to assess the confidence level of the research assistants and whether the instruments correctly measured the symptoms of depression and impact in daily functioning. The research assistants visited each health facility, gauged inclusion/exclusion criteria, obtained written informed consent, and conducted the interviews in a confidential space, either in a spare room within the health facilities or an open ground. Android tablet with a questionnaire application was used for data collection.

### Analysis

Data was collected using an Android tablet with a system in place to minimize missing data and outliers. As a result, there were no missing data points in the dataset. Descriptive statistics were used to report on the socio-demographic characteristics such as age, sex, education, caste/ethnicity, occupation, marital status, religion, number of family members in the household and sufficiency of foods. We presented percentages of the patients who met threshold level for depression based on the Nepali validated cut-off score of PHQ-9 [18], DSM hallmark symptoms (depressed mood or anhedonia) on the PHQ-9 and DSM algorithm. We tested associations between depression with pre-defined risk factors such as age, sex, education, occupation, caste/ethnicity, marital status, number of family members in the household and food sufficiency in the family. We performed bi-variate and multivariate logistic regression to assess the association between depression and socio-demographic and economic characteristics of the participants. The statistical analysis was performed using the Statistical Package for Social Science IBM SPSS-28 [19].

## Results

In total, 1,914 people were approached for participation in the study. 1,897 participants consented to participate and completed the assessments. The majority were female (63.1%). The age of the participants ranged from 18 to 91 years with a mean age of 48.8 years. Most of the participants were between the age of 25 to 59 (58.3%), having secondary or higher level of education (29.5%), currently married (79.6%), and were Brahman/Chhetri (60.8%).

Table 1 shows that the prevalence of depression was higher among female (16.5%), illiterate (17.1%), unemployed (22.6%) and widow/widower/separated (24.5%) participants, as well as those from Janajati (ethnic minority groups, 18.2%); and smaller household size (participants having 1 to 4 members in the family (17.6%).

**Table 1:** Socio-demographic characteristics and percentage who met threshold for depression

Variables	Socio-demographic characteristics of the sample *N* (%)	Percent who met the threshold for depression *N* based on PHQ-9 sum score (%)
**Sex**		
Male	700 (36.9)	78 (11.1)
Female	1197 (63.1)	198 (16.5)
**Age**		
18–24 years	185 (9.8)	31 (16.8)
25–59 years	1106 (58.3)	162 (14.6)
60 and above	606 (31.9)	83 (13.7)
**Education**		
Illiterate/No schooling	345 (18.2)	59 (17.1)
Literate/ less than primary level schooling	496 (26.1)	80 (16.1)
Primary level schooling	496 (26.1)	70 (14.1)
Secondary level schooling or above	560 (29.5)	67 (12.0)
**Occupation**		
Agriculture	798 (42.1)	115 (14.4)
Service/business	404 (21.3)	61 (15.1)
Unemployed	31 (1.6)	7 (22.6)
Other(Students/household works and pension)	664 (35.0)	93 (14.0)
**Caste**		
Brahmin/Chhetri	1154 (60.8)	150 (13.0)
Janajati	435 (22.9)	79 (18.2)
Dalits	153 (8.1)	23 (15.0)
Others	155 (8.2)	24 (15.5)
**Religion**		
Hindu	1690 (89.1)	252 (14.9)
Buddhist	82 (4.3)	10 (12.2)
Others	125 (6.6)	14 (11.2)
**Marital Status**		
Single	191 (10.1)	35 (18.3)
Married	1510 (79.6)	193 (12.8)
Widowed/Divorced/Separated	196 (10.3)	48 (24.5)
**Number of family members**		
1–4 individuals	857 (45.2)	151 (17.6)
5–7 individuals	907 (47.8)	112 (12.3)
> 7 individuals	133 (7.0)	13 (9.8)
**Food sufficiency from family income**		
0–6 months	155 (8.2)	28 (18.1)
6–9 months	226 (11.9)	51 (22.6)
More than 9 months	1516 (79.9)	197 (13.0)
Total	1897 (100.0)	276 (14.5)

### Prevalence of depression

**Fig. 1:**
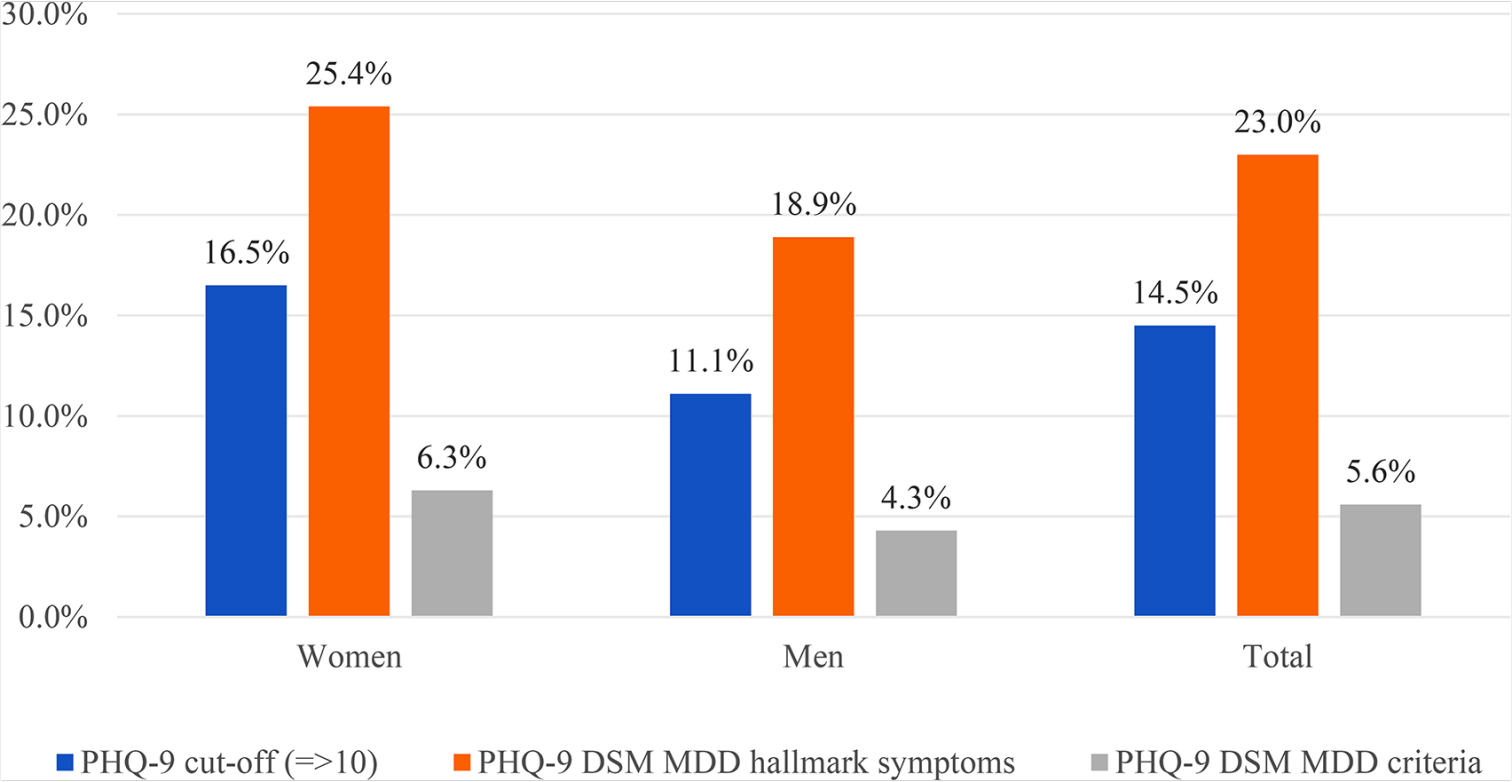
Prevalence of depression

Figure 1 presents the percentage of participants who met threshold for depression based on the locally validated PHQ-9 cut-off sum score, DSM major depressive disorder (MDD) hallmark symptoms and DSM MDD criteria (anhedonia symptoms). The result shows that 14.5% of the participants met threshold for depression based on the PHQ-9 cut-of scores. Hallmark symptoms of depression (depressed mood or anhedonia) were reported by 25.4% of women and 18.9% of men. The prevalence of depression was higher among women in all three measurements i.e. PHQ-9 cut-off (16.5%), hallmark symptoms (25.4%) and DSM-algorithm scoring of PHQ-9 (6.3%).

Table 2 presents item analysis of each PHQ-9 item and DSM hallmark symptoms for male and female participants. The most commonly experienced symptoms (most of the time or always) of depression reported by both male and female patients were little energy (female, 34.1%, and male, 29.3%), sleep difficulties (female, 20.7% and male, 16.6%), and little interest or pleasure/anhedonia (female, 15.1%, and male, 11.7%). These symptoms were significantly more frequent among females. Similarly, DSM hallmark symptoms were also frequent among female patients.

**Table 2:** percentage of participants who endorsed most of time or always in PHQ-9 items

PHQ9 items	Male	Female	Chi-square	*p*-value
PHQ1 (little interest or pleasure)	11.7	15.1	4.29	0.038
PHQ2 (feeling down/depressed/hopeless)	13.7	20.0	11.87	< 0.001
PHQ3 (sleep difficulties)	16.6	20.7	4.90	0.027
PHQ4 (little energy)	29.3	34.1	4.65	0.031
PHQ5 (poor appetite or overeating)	10.1	16.0	12.86	< 0.001
PHQ6 (feeling like failure)	8.7	8.5	0.21	0.885
PHQ7 (trouble concentrating))	5.7	6.3	0.31	0.578
PHQ8 (psychomotor agitation/retardation)	4.7	4.7	0.00	0.972
PHQ9 (suicidality/self-harm)	1.4	1.8	0.45	0.504
DSM Hallmark symptoms (PHQ1 and PHQ2)	18.9	25.4	10.67	< 0.001

Table 3 presents the variables associated with depression in bivariate and multivariate logistic regression models. The prevalence rate of depression varied based on sex, level of education, caste/ethnicity, marital status and number of family members in the household in the bivariate model. Level of education lost its significance level in the multivariate model. Females (OR 1.65) and people from Janajati ethnic minority groups (OR 1.48) had significantly higher risk of depression compared to males and Brahman/Chhetri, respectively. On the other hand, participants who were married (OR 0.57), had 5 to 7 members in the family (OR 0.70) or had more than 7 members in the family (OR 0.54) had a reduced risk for depression (Table 3).

**Table 3:** Variables associated with depression

Socio-demographic characteristics	Bivariate			Multivariate		
	OR (Exp B)	P	95%CI	OR (Exp B)	P	95%CI
**Sex**						
Male(reference group)						
Female	1.581	**0.001**	1.194 − 2.092	1.652	**0.003**	1.191–2.292
**Age**						
18–24 years (reference group)						
25–59 years	0.853	0.457	0.560–1.298	0.853	0.569	0.494–1.474
60 and above	0.788	0.300	0.503–1.236	0.786	0.458	0.417–1.483
**Education**						
No schooling (reference group)						
Literate/ less than primary level schooling	0.932	0.709	0.645–1.348	1.041	0.837	0.708–1.532
Primary level schooling	0.797	0.237	0.546–1.162	0.898	0.620	0.587–1.374
Secondary level or above	0.659	**0.031**	0.451 − 0.962	0.690	0.125	0.429–1.109
**Occupation**						
Agriculture						
Service/business	1.056	0.750	0.754–1.479	1.027	0.889	0.708–1.489
Unemployed	1.732	0.213	0.730– 4.113	1.572	0.326	0.637–3.879
Other(Students/HH works and pension)	0.967	0.825	0.720– 1.299	0.789	0.160	0.568–1.098
**Caste**						
Brahman/Chhetri (reference group)						
Janajati	1.485	**0.009**	1.102 − 2.001	1.480	**0.023**	1.056–2.075
Dalit	1.184	0.486	0.736 − 1.905	0.996	0.988	0.602–1.647
Others	1.226	0.393	0.768–1.958	1.195	0.501	0.711–2.010
**Religion**						
Hindu (reference group)						
Buddhist	0.793	0.500	0.404–1.556	0.586	0.149	0.283–1.212
Others	0.720	0.260	0.406–1.275	0.611	0.114	0.331–1.127
**Marital Status**						
Single (reference group)						
Married	0.653	**0.035**	0.439 –0.971	0.572	**0.028**	0.347–0.942
Widowed/Divorced/Separated	1.446	0.141	0.885–2.360	1.000	0.999	0.537–1.860
**Number of families**						
1–4 individuals (reference group)						
5–7 individuals	0.659	**0.002**	0.506 − 0.858	0.700	**0.011**	0.531–0.923
> 7 individuals	0.507	**0.026**	0.278 − 0.922	0.541	**0.049**	0.294–0.998
**Food Sufficiency**						
0–6 months (reference group)						
6–9 months	1.322	0.288	0.790–2.211	1.269	0.381	0.745–2.162
Greater than 9 months	0.677	0.080	0.438–1.047	0.769	0.267	0.483–1.223

*Note* pseudo R2 for multivariable model = 0.035

### Intra-class correlation

The intra-class correlation coefficient (ICC) for the PHQ-9 was calculated with the health facility as the unit of clustering. The ICC was calculated to inform sample size calculations for determining the number of health facilities and number of participants for evaluating the effectiveness of the e-mhGAP app in a future fully-powered trial. The ICC for PHQ-9 total scores across the ten health facilities with the participants collected at baseline (n=537) was 0.01 (95% CI, 0.00-0.04). 

## Discussion

The results of this study indicate that one in seven people attending primary health care facilities in Jhapa met threshold for depression when using a total sum approach with all items of the PHQ-9 based on a locally validated cut-off. The prevalence of depression using sum scores was significantly higher among females compared to males. The most commonly reported symptoms of depression were low energy, sleep difficulties and lack of interest or pleasure. There was a significant difference in the reported symptoms of depression between males and females with females reporting depressive symptoms more frequently. At least one DSM-5 hallmark symptom (depressed mood or anhedonia) was reported by one out of four women and one out of five men. When using the DSM-5 algorithm for scoring the PHQ-9, the prevalence of depression was approximately one out of 20 patients in primary care. This raises a concern that using a total sum score on a screening tool to make a diagnosis could lead to a three-fold overestimation of the prevalence of depression in primary care.

The prevalence rate of depression reported in our study (14.5%) is consistent with or slightly higher than the rates reported in a recent systematic review of studies conducted with patients in primary care settings in low- and middle-income countries using the same PHQ-9 cut-off score [5]. However the prevalence rate in our study is much lower than the prevalence reported among people attending primary healthcare services in Saudi Arabia [20], Malawi [21], India [22, 23], Nigeria [24] and Sri Lanka [25], all of which use a PHQ-9 sum score approach.

The prevalence of depression in our study is comparable to the prevalence of depression identified among people attending primary care [26] and general adults in Chitwan, Nepal [27]. However, it is much lower than the prevalence reported among populations in Nepal affected by natural disasters [28] and conflict [29–31]. Similarly, the prevalence rate reported in our study is slightly lower or comparable to the prevalence rate reported among a nationally representative sample of the adult population in Nepal [32]. However, our prevalence rate is higher than the prevalence of depression reported in the national mental health survey in Nepal, which was only 2.9%; this national prevalence study used the Mini International Neuropsychiatric Interview (MINI) which was not culturally adapted or clinically validated in Nepal [33]. The discrepancy in reported prevalence rates of depression between our study and the national mental health survey may be attributed to the use of a non-validated tool in the national survey and the study setting differences. Our study is facility-based, whereas the national mental health survey is community-based. Additionally, factors such as sample size, sampling strategies, and cultural sensitivity of the instruments used to assess depression may have contributed to the wide variation in the prevalence of depression in Nepal [34].

There were no significant associations between age, occupation, religion and food sufficiency in the family and the prevalence of depression. The Janajati caste/ethnic group had a significantly higher prevalence of depression compared to Brahman/Chhetri. Similarly, married participants and those with more than five members in the family had a lower prevalence of depression. Female participants (16.5%) had a significantly greater risk of depression than males (11.1%) which is consistent with previous studies conducted with the general population [29, 30, 35] and the population seeking care from primary healthcare facilities in Nepal [26]. The higher prevalence of depression among females could be due to the nature and amount of work females perform. In Nepal, males often do not involve themselves in domestic work while women are expected to look after the family and perform household chores even if they are employed [36]. Our results are consistent with studies conducted among primary healthcare attendees in Delhi and Haryana, India [22, 23], Nigeria [24] and Sri Lanka [37].

Other factors associated with depression were the number of family members, marital status and caste/ethnicity. Married participants had a lower risk for depression which is consistent with a previous study conducted in Chitwan, Tanahu and Dang [29]. Our findings are consistent with the study in Saudi Arabia [20]. There was no significant association between depression and age, occupation and religion of the participants which is consistent with the study conducted among the help-seeking population in Chitwan [26].

The results of this study have several implications for improving the detection and management of depression in primary healthcare facilities in Nepal.

First, the results of this study can be used as baseline data for evaluating the services provided by trained primary health care workers. Similarly, the intra-class correlation coefficient reported in this study can be used to estimate the sample size (i.e., number of health facilities, number of patients) for future randomized controlled trial to evaluate the effectiveness of mobile app-based clinical guides.

Second, the results show that some symptoms of depression included in the PHQ-9 are highly prevalent among participants, and there was a significant difference in reporting those symptoms between males and females. If the mobile app-based clinical guide includes the commonly reported symptoms, this could help to increase patient engagement, overall detection, and the accuracy of detection of depression across all primary healthcare facilities in Nepal. The mobile app should also ensure that primary care workers screen for the hallmark symptoms to avoid over-diagnosis of depression.

Third, prior evidence shows that people with depression are more likely to contact primary healthcare workers rather than mental health specialists [8]. The low detection rate of depression by the trained primary healthcare workers in Nepal could be because of the words used by the healthcare workers during consultations. In our previous study, we found a significant increase in the prevalence of depression after changing the wording in the consent form (i.e. using heart-mind problems instead of mental health problems or mental illness) [38]. The idioms related to mental illness (manasik rog or manasik samasya) are understood as problems associated with the brain-mind, and are often perceived as incurable. Therefore, individuals may be less likely to endorse symptoms out of fear of stigma. On the other hand, the idioms related to the heart-mind (man ko samasya) are understood as something that can be healed and are generally socially acceptable to discuss [39]. Detection of depression might be increased if more culturally acceptable idioms are included in the mobile application.

Finally, the results of this study can be helpful to policy makers responsible for planning and implementing mental health services in primary care. The prevalence rate reported in this study can be used to allocate resources for training and supervision of healthcare workers and procurement of psychotropic medications in different municipalities.

There are several limitations to our study that should be acknowledged. First, the study was conducted in 10 primary healthcare facilities in Jhapa district with high patient flow; therefore, the results may not be generalizable to the entire population of Nepal. Second, the PHQ-9 which was used to screen patients for depression, has been found to have a high rate of false positives (6 false positives for every 10 patients screening positive for depression) [18]. Therefore, the prevalence rate reported in our study may be higher than the actual prevalence in the population. To minimize false positive cases, it is recommended to use tiered algorithms and provide regular clinical supervision to trained primary healthcare workers [18]. Third, our study was conducted during the COVID-19 pandemic, which may have influenced the prevalence of depression. Finally, we relied on self-report measures which may have increased the likelihood of bias. Self-report measures have been shown to predict inflated rates of mental health problems [34].

## Conclusion

Depression symptoms are common among people attending primary healthcare facilities in Nepal. However, the most common symptoms do not always align with the two hallmark criteria. Relying solely on total scores from screening tools like the PHQ-9 may lead to an overestimation of prevalence and false positive diagnoses. Training health workers to first screen for hallmark criteria could improve accuracy and help allocate treatment resources more effectively. Additionally, enhancing the capacity of healthcare providers to identify and manage depression in primary healthcare facilities using a mobile app-based clinical guide may increase the detection rate of depression if the app includes the most commonly reported symptoms of depression by the participants in this study.

## Data Availability

Interested parties may notify the EMILIA (E-mhGAP Intervention guide in Low and middle-income countries: proof-of-concept for Impact and Acceptability) investigators of their interest in collaboration, including access to the data-set analyzed here, through the following email: luitelnp@gmail.com.

## Abbreviations

BHSC: Basic Health Service Center
CHU: Community Health Unit
DHO: District health office
DSM-5: Diagnostic and Statistical Manual
HP: Health Post
LMIC: Low and Middle Income Countries
MDD: Major Depressive Disorder
mhGAP: mental health Gap Action Program
PHCC: Primary Health Care Center
PHQ-9: Patient Health Questionnaire
UHC: Urban Health Center
UK: United Kingdom
WHO: World Health Organization
